# Editorial: AI-Based Computer-Aided Diagnosis and Prognosis for Psychiatric Disorders

**DOI:** 10.3389/fnhum.2022.901525

**Published:** 2022-04-18

**Authors:** Miseon Shim, Do-Won Kim, Seung-Hwan Lee, Han-Jeong Hwang

**Affiliations:** ^1^Industry Development Institute, Korea University, Sejong, South Korea; ^2^Department of Electronics and Information Engineering, Korea University, Sejong, South Korea; ^3^Department of Biomedical Engineering, Chonnam National University, Yeosu, South Korea; ^4^Department of Psychiatry, Ilsan Paik Hospital, Inje University, Goyang, South Korea; ^5^Clinical Emotion and Cognition Research Laboratory, Goyang, South Korea; ^6^Interdisciplinary Graduate Program for Artificial Intelligence Smart Convergence Technology, Korea University, Sejong, South Korea

**Keywords:** psychiatric disorders, machine learning, deep learning, neuroimaging tools, endophenotype biomarkers, computer-aided diagnosis (CAD), computer-aided prognosis (CAP)

Since patients with psychiatric disorders are diagnosed by the interview with clinical experts based on self-assessed symptom scales, a risk of misdiagnosis always exists due to human factors, such as the dissimulation of patients' clinical symptoms or miscommunication between patients and clinicians (Hirschfeld and Vornik, 2003; Stensland et al., 2010). It is important for patients with psychiatric disorders to receive appropriate clinical treatments in a timely manner because a recovery rate decreases as the clinical treatment is delayed (Marín, 2016). For example, a recovery rate is ~60% when patients with major depressive disorders are treated in an optimal time, but if not, the recovery rate decreases to about 17% (Verduijn et al., 2017). However, misdiagnosis can cause treatment failure for patients with psychiatric disorders, leading to the aggravation of their psychiatric symptoms. Therefore, recent researchers have emphasized a necessity to develop assistant diagnosis systems to improve diagnosis accuracy. To this end, objective endophenotype biomarkers reflecting neuropathological traits of psychiatric disorders have been developed using two types of neuroimaging modalities: structural imaging tools [e.g., magnetic resonance imaging (MRI) and computed tomography (CT)] and functional imaging tools [electroencephalography (EEG), magnetoencephalography (MEG), and fMRI]. Moreover, computer-aided diagnosis (CAD) and computer-aided prognosis (CAP) systems have been actively developed using endophenotype biomarkers quantified, where artificial intelligence (AI) algorithms are intensively employed such as conventional machine learning and deep learning methods. Nevertheless, more research should be conducted for enhancing the diagnostic performance of biomarker-based CAD and CAP systems and their reliability because the current state-of-the-art results are not enough to be used in clinical practice.

This Research Topic aimed at sharing the current cutting-edge trends as well as discussing future directions in the research filed of endophenotype biomarker-based CAD and CAP systems for the accurate diagnosis of patients with psychiatric disorders. The specific scopes of the Research Topic are summarized by (1) the development of endophenotype biomarkers using neuroimaging tools such as EEG, MEG, MRI, and fMRI; (2) the development of cutting-edge AI algorithm-based CAD and CAP systems for assisting diagnosis of psychiatric disorders; (3) the development of novel interpretation techniques for endophenotype biomarkers as well as AI-based CAD and CAP systems.

The article entitled “*Large-Scale Brain Functional Network Integration for Discrimination of Autism Using a 3-d Deep Learning Model*” by Yang et al., proposed a novel deep learning algorithm-based CAD system based on neural biomarkers to assist the accurate diagnosis of patients with autism spectrum disorder (ASD). To this end, the authors quantified brain functional networks in terms of eight specific brain functions using resting-state fMRI data, such as the primary visual network, dorsal default mode network, ventral default mode network, precuneus network, sensorimotor network, anterior salience network, left central executive network, and right central executive network. They designed a diagnostic model based on a three-dimensional convolution neural network (CNN) to differentiate ASD patients from healthy controls using pre-quantified brain network architectures. As a result, a diagnostic accuracy of 77.74% was obtained when using the large-scale brain network combining eight different networks, which was higher by approximately 6% than the use of each of specific brain networks. The result indicates that large-scale brain functional networks are promising to serve as reliable biomarkers for diagnosis of ASD.

The article entitled “*Identifying and Predicting Autism Spectrum Disorder Based on Multi-Site Structural MRI With Machine Learning*” by Duan et al., employed machine learning techniques under the unified framework in neuroimaging to identify the neuro-biomarkers of patients with ASD base on individual structural MRI data. To this end, the authors processed three-levels of assessments to enhance the interpretability of the machine learning model, such as model-level for extracting biomarkers, feature-level for identifying important biomarkers of the patients with ASD, and biology-level for proving neuroscientific plausibility of the identified biomarkers. The distinct neuro-biomarkers were identified for patients with ASD based on regional gray matter volume, which significantly differed from typically developing controls. Moreover, the distinct neuro-biomarkers were significantly correlated with patients' symptom scores, such as communication skill score and verbal skill. The interpretable machine learning framework can be used to understand the pathophysiological mechanism of ASD and extend it to other psychiatric disorders.

In the article entitled “*Abnormality of Functional Connections in the Resting State Brains of Schizophrenics*” by Zhu et al., the authors developed a brain network-based CAD system to assist diagnosis of patients with schizophrenia using resting-state EEG data. The authors quantified two different levels of brain networks based on graph theory, i.e., different frequency band-based single-layer networks and multi-layer networks. A machine-learning algorithm based on an ensemble learning method was proposed to classify the patients with schizophrenia and healthy controls. As a result, the highest diagnosis accuracy of 89.38% was obtained when using a single-layer network to differentiate the patients with schizophrenia and healthy controls. Moreover, to generalize the feasibility of the developed CAD system for other psychiatric disorders, the authors applied the same approach to differentiate the patients with schizophrenia and Alzheimer's disease and obtained a maximum classification accuracy of 86.8% when using multi-layer networks. It is expected that the proposed network-based features can be also applied to other psychiatric disorders, such as ASD, bipolar disorder, depression, attention deficit hyperactivity disorder, and so on.

A fourth and final study by Peng et al., performed a systematic review about the significant effect of AI-based brain-computer interface (BCI) training on rehabilitation of post-stroke patients suffering from upper limb dysfunction by analyzing randomized controlled trials. Note that this study was not directly related to the Research Topic, but indirectly related because stroke is one of the major neurological disorders that tend to secondarily result in psychiatric disorders. The authors investigated 16 randomized controlled trials involving 488 participants and evaluated the quality of studies to examine the efficacy of BCI for the rehabilitation using Cochrane's risk of bias tool. From the systematic review, it was found that the BCI-based rehabilitation system can effectively improve the upper limb motor function of post-stroke patients.

In this Research Topic, we provide the three research articles showing recent advances in AI-based CAD and CAP for psychiatric disorders and one indirectly-related review article. Even though only four articles were published in this Research Topic, we believe that this Research Topic contributes to the advancement of AI-based CAD and CAP for psychiatric disorders and the encouragement of further studies for related researchers.

## Author Contributions

MS wrote the article. D-WK reviewed the article. S-HL and H-JH reviewed and edited the article. All authors contributed to the article and approved the submitted version.

## Funding

This work was supported by the Technology Development Program (S3197996) funded by the Ministry of SMEs and Startups (MSS, Korea), by Basic Science Research Program through the National Research Foundation of Korea (NRF) funded by the MSIT (NRF-2020R1A4A1017775 and NRF-2019R1I1A1A01063313).

## Conflict of Interest

The authors declare that the research was conducted in the absence of any commercial or financial relationships that could be construed as a potential conflict of interest.

## Publisher's Note

All claims expressed in this article are solely those of the authors and do not necessarily represent those of their affiliated organizations, or those of the publisher, the editors and the reviewers. Any product that may be evaluated in this article, or claim that may be made by its manufacturer, is not guaranteed or endorsed by the publisher.
